# The C.O.S.'s Protest

**Published:** 1890-10-04

**Authors:** 


### Passing Topics.

### THE C.O.S.'s PROTEST.

When the Charity Organization Society succeeded in obtaining the appointment of the Lords' Select Committee upon Hospitals, it was not unnatural that the institutions should have regarded the prospect with some misgiving. There was the fact that the society, during a long period of its existence, had adopted towards them a more or less unfriendly attitude, and although, like most other societies and individuals, it was not averse from making use of them when occasion arose, a not infrequent occurrence, few who had studied the question could doubt that the work of the hospitals was accorded but scant appreciation.

The opening of the case before the Lords' Committee by the representatives of the society gave point to these views, and the hospitals had full cause to regret that by their own supineness they had allowed an inquiry eminently desirable in itself, and for which public opinion, so far as it is concerned in such matters, was ripe, to assume the character of a cause in which they were made virtually to pose as defendants.

A recollection of these facts imparts importance to the leaderette in the September review of the society, where regret is expressed that "the attention of the Lords' Committee should have been diverted from the broad question of the organization of medical charity to details of domestic management," and the society's aspiration is that they (the Lords' Committee) "will in future resolutely close their ears to everything in the nature of domestic scandal, and confine their attention to the large and important questions they were appointed to consider." This is precisely the appeal we have already ventured to make more than once in this column, and while the society's protests will scarcely prove pleasant reading to those prints and persons who have so sedulously striven to bring discredit upon a valuable institution, we are of opinion that the society has done well to publicly disclaim sympathy for the producers and retailers of the petty personalities over which so much time has been already grievously wasted.

The evidence of this class is not pleasing, but perhaps the most regrettable feature in connection with this subject is that presented by one or two journals, ostensibly devoted to the cause of the hospitals, since the party of malcontent nurses appeared upon the scene. Surely the readers of these publications must be growing tired of stereotyped spitefulness. Now their conductors are wrath with the editor of one of the daily papers for having, as they say, given place to a letter or letters from hospital authorities while rejecting the communications of certain of their assailants. Does not this display of an entire absence of the sense of proportion explain much? It ought to be clear to the most limited experience that neither space nor patience can be found for every unknown scribe. The responsible representatives of great institutions are palpable entities. Is it possible to say the same of every one who would fain rush into print, and whose real name, even if recorded, signifies nothing, and is but a patent of anonymity? If attention is to be demanded of our journals to every discontented nurse or servant, we know not what manner of institution it is which could hope to escape slander and injury.

We have no admiration for "aristocratic matrons," if there be any of that sort, but it must be evident that there is no hospital but must be called upon to rid itself from time to time, by the act of the matron or other responsible official, of inefficient or unsatisfactory employés. The larger the institution the more frequent the occasion, and the knowledge of human nature must be elementary indeed to suppose that many of those whose resignation is not entirely voluntary are intelligent and magnanimous enough to recognize their own deficiencies. The probability is that most of them nurse a grievance.

Was it to give voice to these the Lords' Committee was appointed? The C.O.S. says no, and in this matter the C.O.S. rightly decides. We trust to the Lords when they meet again to protect themselves against being made a medium for the dissemination of personal grievances and the indulgence of personal rancour. Great questions await their examination; let not these be lost sight of in order that Grub Street may hold carnival. Agricola.

